# Controlled Release of DEET Loaded on Fibrous Mats from Electrospun PMDA/Cyclodextrin Polymer

**DOI:** 10.3390/molecules23071694

**Published:** 2018-07-11

**Authors:** Claudio Cecone, Fabrizio Caldera, Francesco Trotta, Pierangiola Bracco, Marco Zanetti

**Affiliations:** 1 Department of Chemistry and NIS Centre, University of Turin, Via P. Giuria 7, 10125 Torino, Italy; claudio.cecone@unito.it (C.C.); fabrizio.caldera@unito.it (F.C.); francesco.trotta@unito.it (F.T.); 2 ICxT Centre, University of Turin, Lungo Dora Siena 100, 10153 Torino, Italy

**Keywords:** *N*,*N*-diethyl-3-toluamide (DEET), insect repellent, beta-cyclodextrin-based polymer, fibrous nanosponge, controlled-release system

## Abstract

Electrospun beta-cyclodextrin (βCD)-based polymers can combine a high surface-to-volume ratio and a high loading/controlled-release-system potential. In this work, pyromellitic dianhydride (PMDA)/βCD-based nanosponge microfibers were used to study the capability to host a common insect repellent (*N*,*N*-diethyl-3-toluamide (DEET)) and to monitor its release over time. Fibrous samples characterized by an average fibrous diameter of 2.8 ± 0.8 µm were obtained and subsequently loaded with DEET, starting from a 10 g/L diethyl ether (DEET) solution. The loading capacity of the system was assessed via HPLC/UV–Vis analysis and resulted in 130 mg/g. The releasing behavior was followed by leaving fibrous DEET-loaded nanosponge samples in air at room temperature for a period of between 24 h and 2 weeks. The releasing rate and the amount were calculated by thermogravimetric analysis (TGA), and the release of the repellent was found to last for over 2 weeks. Eventually, both the chemical composition and sample morphology were proven to play a key role for the high sample loading capacity, determining the microfibers’ capability to be applied as an effective controlled-release system.

## 1. Introduction

Intensification of human travel and transcontinental commerce are the main causes related to the spreading of most of the emerging infectious diseases. This threat comes from zoonotic viruses transmitted to humans by hematophagous insects such as mosquitoes, sandflies, midges, and ticks, and the diseases are thus designated as arthropod-borne viruses (arboviruses) [1]. Arboviral infections can range from asymptomatic to fulminant fatal diseases and can be caused by at least 135 arboviruses. In most cases, the occurrence of a major outbreak is due to the introduction of viruses in new geographic areas where the hosts are susceptible and able to sustain infection [2]. Lyme disease; Heartland virus disease; borreliosis; and 346 rickettsiosis (tick-borne), dengue, and chikungunya (mosquito-borne) diseases are some of the most recent diseases described in the United States and Europe. With few exceptions, these infectious diseases cannot be prevented by vaccinations [3]. In order to prevent insects landing and biting, effective preventive measures are window screens and insect-repellent formulations in buildings and open environments. Repellents are natural or synthetic chemical substances applied to clothing or skin; the main vehicles for these formulations are sprays, lotions, and gels. Among the synthetic repellents, *N*,*N*-diethyl-3-toluamide (DEET), ethyl butylacetylaminopropionate (EB), and 1-(1-methylpropoxycarbonyl)-2-(2-hydroxyethyl) (picaridin or icaridin) are the most widely used [4]. Despite that its discovery dates back to 1946, DEET remains the most popular and successful product. It is a yellowish, oily, volatile liquid (at room temperature) that is characterized by an unpleasant odor and is moderately soluble in petroleum ether, insoluble in water, and very soluble in alcohol. DEET is incorporated into topical formulation from 7 wt % (short repellent action, up to 2 h) to 30 wt % (long period action, up to 6 h) [5]. With proper application, the safety record of DEET has proven to be excellent, with most of the toxicity cases confined to children after overapplication or ingestion [3]. However, at high concentrations and after prolonged exposure, adverse effects have been reported, caused by the presence of DEET in the bloodstream. As a result of its lipophilic feature, it can be absorbed by the skin and pass through the cutaneous barrier, reaching deeper layers and blood vessels [6]. Skin rash, seizures, encephalopathy, and central nervous system toxicity have been reported [7,8,9]. Moreover, DEET has also been found to damage synthetic fabrics, leather, plastics, and painted surfaces [3,7]. One effective strategy to avoid DEET-based-formulation drawbacks could be loading the molecule into a proper absorbent system, such as a high-surface-to-volume-ratio matrix. In this way, the contact between the repellent and both skin and fabrics would be minimized. A fibrous morphology would increase the surface-to-volume ratio, and the choice of suitable materials would enhance the repellent loading capacity.

Beta-cyclodextrins (βCDs), cyclic oligosaccharides obtained enzymatically from starch, are composed of seven α-(1,4)-linked glucopyranose units, arranged surrounding a slightly lipophilic cavity in a peculiar truncated-cone-shaped structure. Because of the presence of a lipophilic domain, βCDs can accommodate guest molecules through the formation of inclusion complexes [10]. Additionally, because of the presence of a high number of hydroxyl functionalities, βCDs can be used as building blocks for several polymer synthesis techniques. Several classes of di-, tri- or tetra-functional molecules, such as carbonyldiimidazole, pyromellitic dianhydride (PMDA), hexamethylene diisocyanate, and citric acid, have been reported as βCD linking molecules [11]. By choosing proper synthetic conditions, both water-soluble (hyper-branched) and water-insoluble (cross-linked or nanosponge) polymers have been successfully obtained [12]. As a result of their tuneable syntheses, these types of polymers are adopted in several applications, such as for drug carriers, gas traps, fillers, cosmetic photo-stabilizers, metal cation absorbers, and fire-retardant agents [13,14].

The DEET loading capacity and controlled release of a βCD-grafted cotton fabric have been previously reported [15]. However, the system described displayed weak repellency effects against mosquitoes. This behavior was related to a slow DEET volatilization from the matrix, likely as a result of a small loaded quantity and a strong interaction between the repellent and fabric. Unlike that reported for a βCD-grafted fabric, a βCD-based polymer, synthesized using βCD as the polymer building block, would be characterized by a higher number of hosting sites. Thus, a higher DEET loading capacity and higher releasing rates would be expected. Moreover, this kind of DEET-loaded matrix may result in a prolonged and controlled-release system and may eventually be helpful to minimize toxicity, health problems, and fabric-damage issues.

Electrospinning is a powerful technique that allows microfiber and nanofiber deposition from a material dissolved in a solvent through an electrically forced fluid jet [16]. Filtration, tissue engineering, biosensors, drug delivery, wound dressings, and enzyme immobilization are just a few of the applications covered by electrospun fibers [17]. As a result of an electric field applied between a nozzle and a collector, stretching (towards the collector) of a polymeric solution (or melt) that flows out of the nozzle is induced. A volumetric pump keeps a constant flow while the field is managed by an electric field generator. Micrometric dimensions and a high surface-to-volume ratio of the produced fibrous mats, as well as simplicity, versatility, and cost-efficiency, are the main features of this technique [18].

Obtaining fibers starting from a water-soluble βCD-based polymer would offer the possibility to obtain a high-surface-to-volume-ratio product, capable to entrap guest molecules and release them over the time. We recently demonstrated the possibility to use a simple thermal treatment to convert βCD-based fibers, electrospun from a water solution, into a water- and organic-solvent-insoluble mat [19].

In the present study, we investigated the capability of such fibrous βCD-based nanosponges to host chemicals, in this case, a common insect repellent (DEET), and to gradually release them over time. The fibrous nanosponges were obtained by thermally treating an electrospun mat, processed starting from a water-soluble βCD/PMDA polymer.

## 2. Experimental Section

### 2.1. Materials

Dimethyl sulfoxide (DMSO), triethylamine, PMDA, ethyl acetate, DEET, and ethyl ether were purchased from Sigma-Aldrich (Darmstadt, Germany) and were used as received. βCD was provided by Roquette Italia (Cassano Spinola, Italy) and was dried in an oven at 100 °C up to a constant weight before use. The cotton fabric used was a lint-free 100% medium-weight cotton twill (5 oz) from the Agilent GC-MS (Santa Clara, CA, USA) cleaning kit; as was the cotton swab.

### 2.2. Polymer Synthesis

The polymer synthesis was performed following the procedure described in the work of Trotta et al. (2014) [20]. Briefly, 6 mL of DMSO was used to dissolve 0.977 g (0.86 mmol) of anhydrous βCD. Subsequently, 1 mL (7.17 mmol) of TEA was added as a catalyst, followed by 2.254 g (10.33 mmol) of PMDA introduced under vigorous stirring at room temperature. This step was related to a solution viscosity increase. After 24 h, an excess of ethyl acetate was used to precipitate and wash the product from the reactive media, which was recovered by vacuum filtration. Afterwards, the dry product was solubilized in deionized water, freeze-dried, and finally stored in a desiccator. Considering the weight of the freeze-dried polymer with respect to the theoretical weight, equal to the sum of βCD and PMDA, the yield result was approximately 90%.

### 2.3. Polymer Processing and Curing

The polymer processing and its thermal treatment were performed according to the work of Cecone et al. (2018) [19]. In short, a 59 wt % distilled water/polymer solution was chosen as the optimum fiber-forming concentration. The electrospinning was performed by setting the working distance to 15 cm, the field strength to 30 kV, and the flow to 1.2 mL/h. The thermal treatment was performed at 180 °C under nitrogen flow with a 10 °C/min heating rate, followed by 10 min isotherm.

### 2.4. Fibrous-Nanosponge DEET Loading and Extraction Procedures

DEET loading was performed using 5 mL of a 10 g/L diethyl ether (DEET) solution per loading. Each substrate (100 mg) was left in the solution for 24 h and was then recovered, dried, and stored. Before loading, each substrate was dried in an oven at 70 °C.

DEET extraction from the loaded polymer was performed using a methanol/water (57/43 *vol*/*vol*) mixture. Eight extractions were set, starting from 10 mg of loaded polymer. Except for the last extraction, which lasted 24 h without additional treatments, each extraction lasted 30 min under sonication at room temperature, using 1 mL of extracting mixture each. All the extracted fractions were separated from the solid by centrifugation, recovered, and stored in a freezer. After each extraction, fresh mixture was used to start the next.

### 2.5. Cotton Samples’ Loading Procedure

In order to compare the loading capacity of the βCD polymer with that of conventional natural fabrics, two cotton samples, in the form of a swab and fabric, were also loaded with DEET. The loading step was performed following the procedure described above for the fibrous nanosponges.

### 2.6. Nanosponge-Powder Loading Procedure

In order to investigate the effect of the fibrous morphology on the loading capacity of the system, the same βCD-based polymer, cured without being processed into fibers (polymer powder), was also loaded with DEET. The loading step was performed following the procedure described above for the fibrous nanosponges.

### 2.7. Fibrous-Nanosponge DEET Release Study Setup

In order to evaluate the DEET release rate from the matrix, five samples of 10 mg each were prepared from the spun DEET-loaded mat. These samples were then left in air and at room temperature for 24 h, 48 h, 72 h, 1 week, and 2 weeks, and a thermogravimetric analysis (TGA) was performed at each time interval.

### 2.8. Scanning Electron Microscopy Analysis

The morphological features of the fibers were checked using scanning electron microscopy (SEM). The images were acquired with a Leica Stereoscan 410 Oxford Instrument (Abingdon, UK), using secondary electrons and a 10 kV accelerating voltage.

### 2.9. Thermogravimetric Analysis Measurements

TGA was carried out using a TA Instruments Q500 TGA (New Castle, DE, USA), from 50 to 400 °C, under nitrogen flow, and with a heating rate of 10 °C/min.

### 2.10. HPLC Measurements

A Perkin-Helmer (Waltham, MA, USA) Flexar liquid chromatograph (HPLC) was used to check the total amount of DEET released by the loaded polymer after the extractions.

A 1 to 100 ppm calibration curve was constructed before the sample analysis. A C-18 column was installed for the measure, and a 1 mL/min methanol/water (57/43 *vol*/*vol*) flow was used as the mobile phase. The released DEET, as well as the DEET used for the calibration, were measured with a UV–Vis detector at 220 nm. The duration of a single run was 15 min, with a DEET retention time of 11 min.

## 3. Results and Discussion

### 3.1. Polymer Processing: Analysis of the Morphology

**Figure 1:**
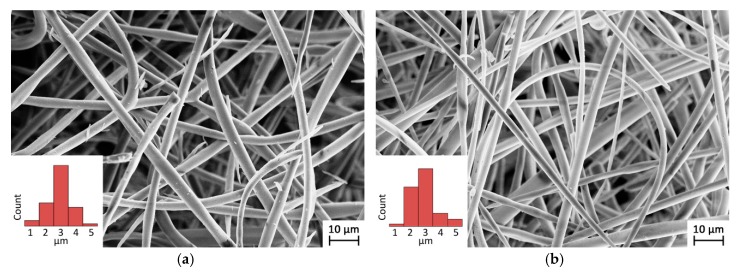
Scanning electron microscopy (SEM) image and fiber diameter distribution of the (**a**) as-spun polymer, and (**b**) thermal-treated sample.

Figure 1 reports the comparison between the as-spun mat (a) and a mat obtained after the spinning process and thermal treatment at 180 °C (b). The SEM examination confirmed that the fibrous morphology displayed by the as-spun sample (average fiber diameter of 2.9 ± 0.7 µm) was retained after the curing process (average fiber diameter of 2.8 ± 0.8 µm).

### 3.2. DEET Loading Capacity Assessment

Subsequently to the DEET loading process, the absorbed repellent was extracted in 8 mL of a methanol/water (57/43 *vol*/*vol*) mixture, and the total amount was estimated via HPLC/UV–Vis. Four consecutive extractions (1 mL each) of the same sample resulted in enough to assess a loading capacity of 130 mg/g.

The loading capacity obtained for this system represents an interesting result, particularly when compared to the results reported in the study by Peila et al. (2017) [21], in which the best-performing system, a polyester incorporated with βCD, was characterized by a DEET loading capacity of 29 mg/g.

### 3.3. Thermogravimetric Analysis

**Figure 2:**
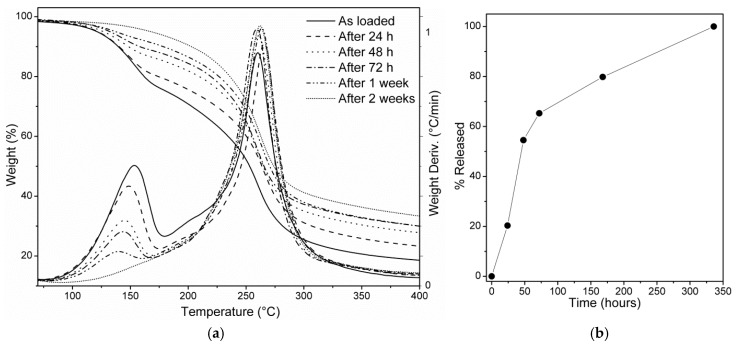
(**a**) Thermogravimetric analysis (TGA) of the as-loaded polymer 24 h, 48 h, 72 h, 1 week, and 2 weeks after the loading step, respectively. (**b**) DEET releasing profile.

Additionally, a TGA of the loaded mat was performed and is reported in Figure 2a (solid line). Two different weight-loss phenomena were observed. The first, occurring approximately between 100 and 180 °C, with a weight-loss rate maximum of close to 160 °C represented repellent volatilization from the matrix. The second, occurring above 200 °C, was related to the thermal degradation of the nanosponges. The amount of DEET loaded into the fibers was then estimated through the weight loss observed between 100 and 180 °C. Following this approach, the loading capacity of the system was 173 mg/g, thus being higher than that obtained via HPLC/UV–Vis. However, the thermogram showed that the weight-loss phenomena related to the DEET volatilization and to the nanosponge pyrolysis respectively were not well separated, as the end of the first overlapped the early stages of the second (Figure 2a, solid line). This overlap may have resulted in an overestimation of the system loading capacity calculated via the gravimetric approach.

### 3.4. DEET Release Study

The overlap of the thermograms along with their respective derivative curves, resulting from the release study of DEET from the loaded matrix, are reported in Figure 2a. The comparison shows that the amount of loaded DEET decreased over time, indicating that the repellent gradually volatilized from the matrix. This decrease was more apparent in the derivatives’ overlap, in which the intensity of the peak related to the DEET volatilization decreased by increasing the time the sample was left in open air. After 2 weeks of exposure, the weight loss related to DEET volatilization became negligible, indicating little or no residual DEET still loaded in the matrix. By assuming the 2 weeks (*t*_2 *weeks*_) old sample as the 100% DEET-released scenario and the as-loaded (*t*_0_) sample as the 0% DEET-released scenario, the fraction of DEET released after each time interval (*t_x_*) was estimated as follows and is reported in Figure 2b:

(1) $$DEET\;wt\;\;\%\;released\;at\;t_{x}=\frac{\left(Wt_{x}-\;Wt_{0}\right)}{\left(Wt_{2\;weeks}-Wt_{0}\right)}*100.$$

where *Wt_x_* is the loaded DEET after each time interval *t_x_*, *Wt*_0_ is the loaded DEET after the loading step (0% DEET-released scenario), and *Wt*_2 *weeks*_ is the 100% DEET-released scenario.

The release profile (Figure 2b) showed a rapid release over the first 72 h, which consisted of more than 60% of the loaded DEET, followed by a slow release of the remaining amount over the next 11 days.

Host–guest complexes between βCD and a large number of chemicals have been studied in the past decades [22]. Recently, it has been demonstrated how cyclodextrins appear to affect DEET formulation by complexation and emulsion stabilization, without negatively affecting its volatility [23]. Furthermore, tests carried out on βCD-grafted fabrics have already been reported [15,21]. Thus, considering the polymer chemical composition and its molecular arrangement, two types of molecular interactions between the sample and the DEET molecules could be hypothesized: one consisting of host–guest inclusion complexes expressed by the βCD molecules composing the polymer; additionally, another interaction expressed by the sterical entrapment of the repellent within the cross-linked polymer chains. The two different interactions could explain the release profile. The first release, occurring during the first 72 h, may have been due to the molecules entrapped within the polymer network, which were less retained and thus free to volatilize rapidly. Vice versa, the complexed DEET molecules could justify the slower release that occurred over the remaining 11 days, which consisted of less than 40% of the total amount.

**Figure 3:**
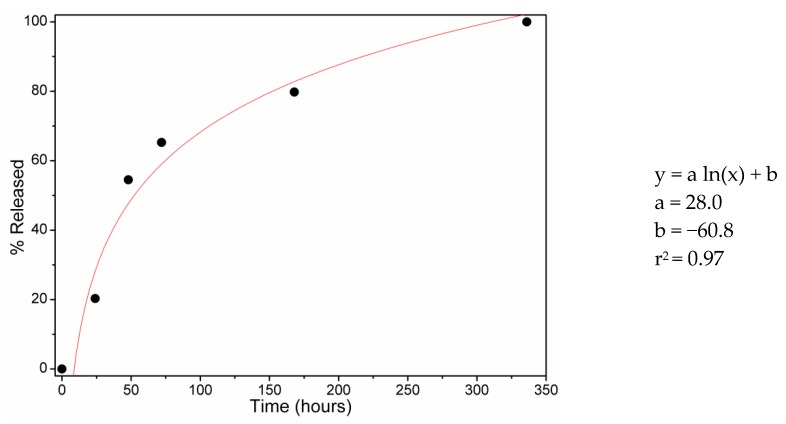
Logarithm fit of DEET releasing profile.

Nevertheless, as it is possible to observe from Figure 3, the releasing profile could also be easily fitted with a logarithm curve. Given this result, the volatilization of DEET from the matrix could also be described by the following equation:

(2) $$d\left[Q_{DEET}\right]/dt=k\left[Q_{DEET}\right],$$

in which the volatilization rate was dependent only on the quantity of loaded DEET ([*Q_DEET_*]).

### 3.5. DEET Loading Capacity with Respect to the Morphology and Chemical Composition

**Figure 4:**
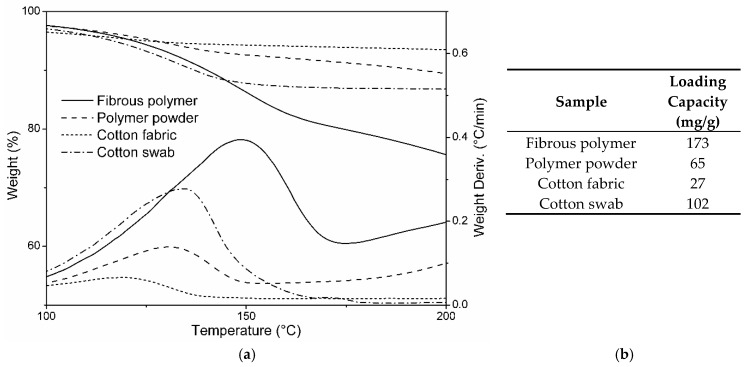
(**a**) Thermogravimetric analysis (TGA) and (**b**) DEET loading capacity of beta-cyclodextrin (βCD)-based fibrous polymer (Fibrous polymer), the same polymer but with powder morphology (Polymer powder), a pure cotton fabric (Cotton fabric), and a cotton swab (Cotton swab).

The thermograms reported in Figure 4 show how the chemical nature of the βCD-based polymer deeply affected its capability to load guest molecules, when compared to a standard fabric-like cotton, which showed an almost negligible loading. A higher loading capacity, approximately 4 times higher than that of the cotton fabric, was displayed by the cotton swab. This result was likely due to its less compact structure compared to the cotton fabric. However, the loading capacity displayed by the cotton swab was still lower when compared to that of the βCD polymer sample. Moreover, given the absence of strong intermolecular interactions with this kind of sample, a fast DEET release due to volatilization was also expected.

The comparison between the TGA of both βCD-based polymers (Figure 3) demonstrated that the fibrous morphology played a key role in increasing the loading capacity. This observation reflects the results reported in the work of Peila et al. (2017) [21]. In that work, βCD-based powder samples (comparable to the polymer powder reported in this work) were loaded with DEET and then incorporated into polyester fabrics. The loading capacity observed resulted in the same magnitude of the polymer powder.

It was also apparent that, for the fibrous polymer, the weight-loss rate maximum (associated to DEET volatilization) was shifted towards higher temperatures with respect to both the polymer powder and cotton samples. This behavior could be related to the capability of the fibrous βCD-based polymer to create stronger interactions with DEET molecules.

A high surface-to-volume ratio typical of the fibrous samples (absent in the polymer powder) and a chemical structure capable to accommodate guest molecules, typical of βCD-based products (absent in standard fabrics), were both present in this system and were demonstrated to deeply affect the system performance.

## 4. Conclusions

A water-soluble βCD-based polymer was obtained using PMDA as a linking molecule and working under critical dilution conditions. Because of the polymer solubility features, an electrospinning technique was used to produce fibrous mats, using distilled water as the unique solvent, while a subsequent one-step thermal treatment (of the spun mat) allowed fibrous βCD-based nanosponges to be obtained. The final sample morphology was characterized by well-defined fibers with a fiber diameter distribution of 2.8 ± 0.8 µm. 

Because βCD-based nanosponges are known as a powerful tool for the controlled release of drugs and chemicals, the loading capacity and the controlled release of the obtained fibrous nanosponges towards a common insect repellent (DEET) were studied. 

HPLC/UV–Vis was used to assess the DEET loading capacity, which was found to be 130 mg/g, while the release of the repellent from the matrix was followed via gravimetric analysis and lasted for over 2 weeks. From the releasing profile, it was possible to hypothesize two loading mechanisms consisting of host–guest complexes and steric entrapment, and as a result of the successful logarithm fit of the releasing profile, it was also possible to demonstrate how the volatilization of DEET from the matrix was dependent on the loaded DEET amount.

Comparisons with a cotton fabric and a cotton swab, loaded following the same procedure as for the βCD-based nanosponges, demonstrated how the chemical composition of the βCD-based nanosponges was an essential feature in order to reach high loading capacities. Eventually, it was proven how the sample morphology also affected the high DEET loading capacity of the system, by comparing the spun polymer with the same polymer matrix but in powder form, loaded the same way as for the fibrous mat.

The results obtained can be considered an encouraging starting point for further works, as the system fully demonstrated the capability to host DEET molecules and provide a gradual release over time.
